# Health Tract, No. 174

**Published:** 1865

**Authors:** 


HEALTH TRACT, No. 174.

### PHILOSOPHY OF EXERCISE.

All know that the less we exercise the less health we have, and the more certain are we to die before our time. But comparatively few persons are able to explain how does exercise promote health. Both beast and bird, in a state of nature, are exempt from disease, except in rare cases; it is because the unappeasable instinct of searching for their necessary food, impels them to ceaseless activities. Children, when left to themselves, eat a great deal and have excellent health, because they will be doing something all the time, until they become so tired they fall asleep; and as soon as they wake, they begin right away to run about again; thus their whole existence is spent in alternate eating, and sleeping, and exercise, which is interesting and pleasurable. The health of childhood would be enjoyed by those of maturer years, if, like children, they would eat only when they are hungry; stop when they have done; take rest in sleep as soon as they are tired; and when not eating or resting, would spend the time diligently in such muscular activities as would be interesting, agreeable, and profitable. Exercise without mental elasticity, without an enlivenment of the feelings and the mind, is of comparatively little value.

1. Exercise is health-producing, because it works off and out of the system its waste, dead, and effete matters; these are all converted into a liquid form, called by some "humors," which have exit from the body through the "pores" of the skin in the shape of perspiration, which all have seen, and which all know is the result of exercise, when the body is in a state of health. Thus it is, that persons who do not perspire, who have a dry skin, are always either feverish or chilly, and are never well, and never can be as long as that condition exists. So exercise, by working out of the system its waste, decayed, and useless matters, keeps the human machine "free;" otherwise it would soon clog up, and the wheels of life would stop forever!

2. Exercise improves the health, because every step a man takes tends to impart motion to the bowels; a proper amount of exercise keeps them acting once in every twenty-four hours; if they have not motion enough, there is constipation, which brings on very many fatal diseases; hence exercise, especially that of walking, wards off innumerable diseases, when it is kept up to an extent equal to inducing one action of the bowels daily.

3. Exercise is healthful, because the more we exercise the faster we breathe. If we breathe faster, we take that much more air into the lungs; but it is the air we breathe which purifies the blood, and the more air we take in, the more perfectly is that process performed; the purer the blood is, and as every body knows, the better the health must be. Hence, when a person's lungs are impaired, he does not take in enough air for the wants of the system; that being the case, the air he does breathe should be the purest possible, which is out-door air. Hence, the more a consumptive stays in the house, the more certain and more speedy is his death.

